# Salicylic Acid Treatment and Its Effect on Seed Yield and Seed Molecular Composition of *Pisum sativum* under Abiotic Stress

**DOI:** 10.3390/ijms24065454

**Published:** 2023-03-13

**Authors:** Veronika Berková, Miroslav Berka, Michaela Kameniarová, Romana Kopecká, Marharyta Kuzmenko, Šarlota Shejbalová, Dmytro Abramov, Petr Čičmanec, Lucie Frejlichová, Novák Jan, Břetislav Brzobohatý, Martin Černý

**Affiliations:** 1 Department of Molecular Biology and Radiobiology, Faculty of AgriSciences, Mendel University in Brno, 61300 Brno, Czech Republic; 2 Mendeleum—Institute of Genetics, Faculty of Horticulture, Mendel University in Brno, Valtická 334, 69144 Lednice na Moravě, Czech Republic

**Keywords:** seed development, proteome, heat stress, lipidome, metabolome, field, phytohormone, stress attenuation, yield

## Abstract

The reproductive stage of plant development has the most critical impact on yield. Flowering is highly sensitive to abiotic stress, and increasing temperatures and drought harm crop yields. Salicylic acid is a phytohormone that regulates flowering and promotes stress resilience in plants. However, the exact molecular mechanisms and the level of protection are far from understood and seem to be species-specific. Here, the effect of salicylic acid was tested in a field experiment with *Pisum sativum* exposed to heat stress. Salicylic acid was administered at two different stages of flowering, and its effect on the yield and composition of the harvested seeds was followed. Plants treated with salicylic acid produced larger seed pods, and a significant increase in dry weight was found for the plants with a delayed application of salicylic acid. The analyses of the seed proteome, lipidome, and metabolome did not show any negative impact of salicylic treatment on seed composition. Identified processes that could be responsible for the observed improvement in seed yields included an increase in polyamine biosynthesis, accumulation of storage lipids and lysophosphatidylcholines, a higher abundance of components of chromatin regulation, calmodulin-like protein, and threonine synthase, and indicated a decrease in sensitivity to abscisic acid signaling.

## 1. Introduction

Field pea (*Pisum sativum* L.) belongs to the cool-season food legumes with a heat stress temperature threshold of approximately 25 °C, and daily temperatures above this limit often result in a decrease in seed production [1]. Heat stress during the reproductive stage is the most detrimental. High temperature negatively affects flower initiation, pollen viability, stigma receptivity, ovule viability, ovule size, fertilization, seed set, and seed quality [2,3,4]. In effect, an increase in the maximum daily temperature above 30 °C may result in a reduction in seed yield higher than 50% [5].

Flowering is a crucial developmental transition that results from coordinated interactions of a large number of factors. These factors integrate internal signals and external stimuli, and it is a period highly sensitive to stress [6]. Plants’ response to stressors may vary depending on the species and severity of the stress, but in general, flowering time can either be accelerated or delayed [7]. Salicylic acid is one of the transmissible signals that are produced to induce flowering under stress conditions. It is a prominent stress response phytohormone critical for both biotic and abiotic stress responses. Salicylic acid regulates the transition to flowering by regulating key floral genes, including *FLOWERING LOCUS C*, *CONSTANS*, and *SUPPRESSOR OF OVEREXPRESSION OF CONSTANS 1*, and *FLOWERING LOCUS T* [6,8,9,10]. Salicylic acid is also required for the activation of systemic acquired resistance, which has a common regulatory mechanism with flowering activation [11]. Furthermore, it plays an important role in the regulation of pollen viability, leaf morphology, biomass production, chlorophyll content, and antioxidant enzyme activity under heat stress [12,13,14,15,16].

Previous experiments collectively indicated that salicylic acid could promote tolerance to heat stress and increase the yields of crop plants. Here, the effects of salicylic treatment on *P. sativum* yield were compared in a field experiment. The seeds were sown approximately 60 days after the recommended optimal date to ensure the exposure of the plants to heat stress during the reproductive stage. The ripe seed pods were then collected, and the yield, seed proteome, lipidome, and metabolome were analyzed.

## 2. Results

### 2.1. Field Experiment Results

**Figure 1:**
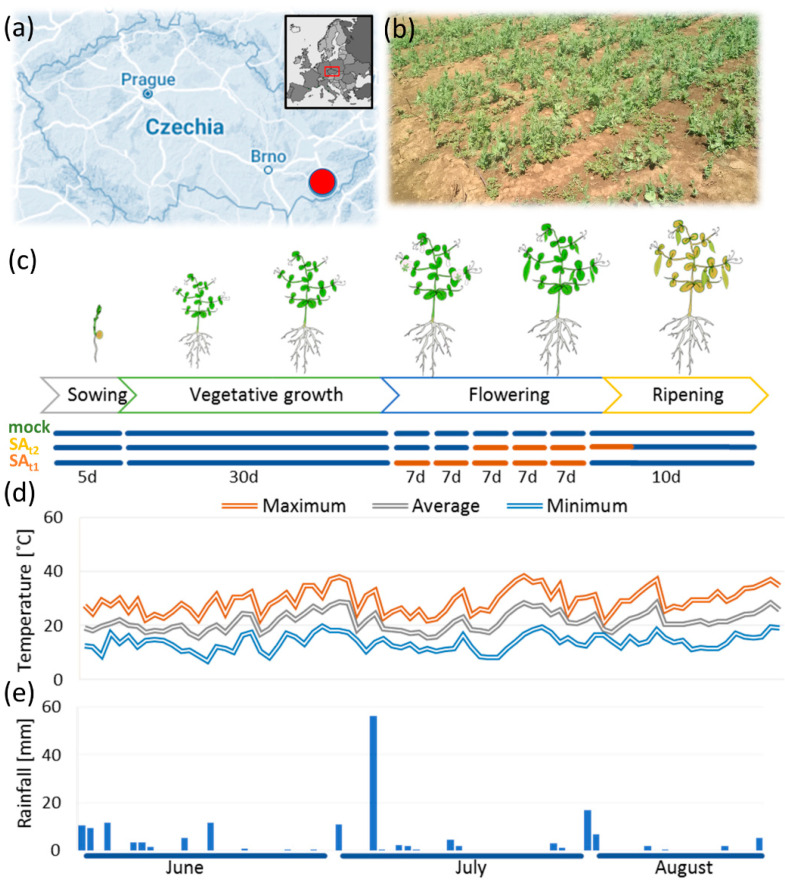
Experimental design. (**a**) Geographical location of the experimental field; (**b**) Field experiment, vegetative growth stage; (**c**) Experimental design. Orange blocks represent treatment with 100 µM salicylic acid; (**d**,**e**) Temperature and rainfall profile.

The experiment was carried out in a dedicated private experimental field in the south of Moravia (Figure 1a,b), and 300 seeds were sown in three isolated blocks to mitigate the possibility of salicylic acid spillage between treated and control plants. The sowing time was selected based on long-term temperature averages to expose flowering plants to heat stress. The application of fertilizers, pesticides, and fungicides was avoided to prevent undesirable side effects in the experiment. Plants were regularly monitored, and those that manifested signs of growth defects or infection were removed from the field. Sufficient rainfall and optimal temperatures promoted vegetative growth, and most plants reached the flowering stage after one month of growth. Next, two sets of plants designated SA_t1_ and SA_t2_ were sprayed weekly with 100 µM salicylic acid starting before the onset of flowering and two weeks later, respectively (Figure 1c). The average maximum temperature during flowering was about 29.6 ± 4.9°, and the heat waves were accompanied by rainfall scarcity, with the last significant rainfall occurring before the onset of flowering (Figure 1d,e). Treatment did not manifest any striking differences in the overall fitness of plants compared to the mock-treated controls.

### 2.2. Salicylic Acid Treatment Increased the Mean Size of Seed Pods

**Figure 2:**
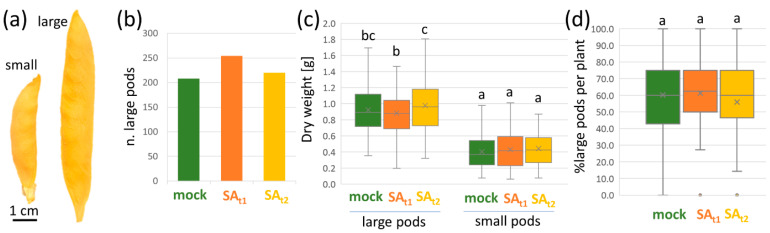
Salicylic acid promoted the growth of seed pods. (**a**) Representative images of collected seed pods classified as small and large (more than 6 cm in length); (**b**) Number of seed pods classified as large; (**c**) Box plot representations of seed weight per pod; (**d**) Percentages of large seed pods per plant including median (line) and mean values (cross). Data are based on 75 plants per experimental condition; the letters represent significant differences (*p* < 0.05, Kruskal–Wallis and Conover’s test, *p* < 0.05).

In total, 75 plants were analyzed per experimental condition. The seed pods were collected and sorted into two categories according to the length of the seed pod. The treatment with salicylic acid positively affected the number of large seed pods per plant, with an increase compared to the mock of 22 and 6% for SA_t1_ and SA_t2_, respectively (Figure 2a). Interestingly, the proportion of large seed pods per plant was not significantly different, with mean values of 60.2 ± 27.4, 61.3 ± 16.9, and 55.9 ± 21.6% for mock, SA_t1_, and SA_t2_, respectively (Figure 2d). The dry mass of the seeds per pod showed that the size of the pod did not fully correlate with the yield (Figure 2b). The highest mean weight of large pods was found for SA_t2_ seed pods, representing a 1.06 and 1.22 increase compared to mock (*p* < 0.1, Student’s *t*-test) and SA_t1_ (*p* < 0.01), respectively. The differences in the mean dry weight of the small pods were not statistically significant (*p* < 0.05, Figure 2d).

### 2.3. Seed Proteome Analysis Did Not Show Striking Differences in Total Proteome Composition

**Figure 3:**
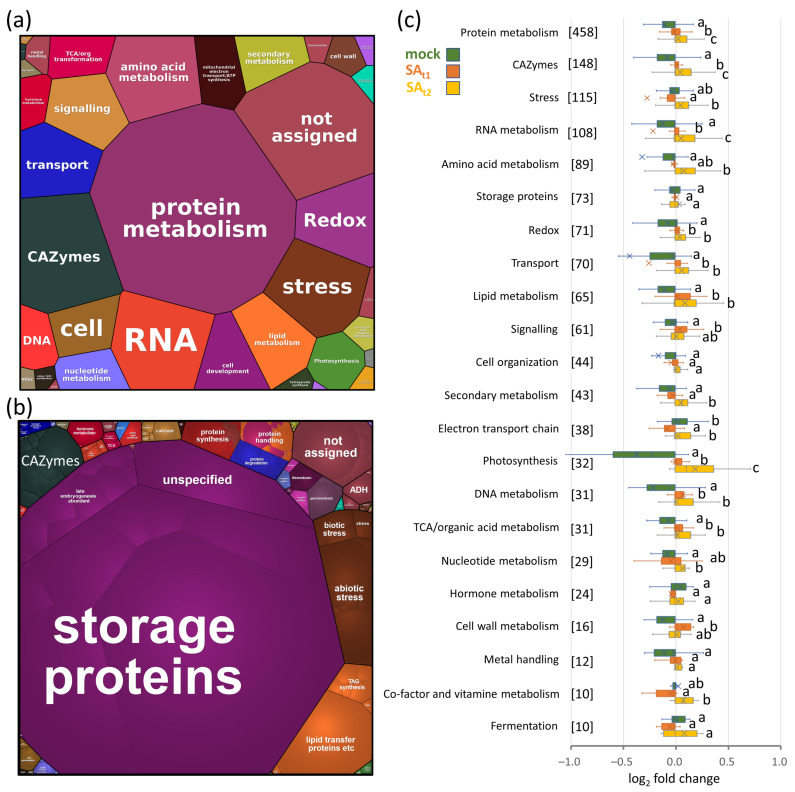
The composition of seed proteome. (**a**,**b**) Visualization of the seed proteome in the ProteoMap based on 1796 quantified proteins and the annotations of their corresponding *Arabidopsis* orthologs. (**a**) Expected functions of quantified proteins. The category size is proportional to the number of identified proteins. (**b**) Estimated protein content in the seeds of mock-treated plants. The category size is proportional to the estimated protein abundance. (**c**) Expected effects on 22 metabolic pathways based on the observed differences in protein abundance. Box plot representation of log2 fold changes in protein abundances including median (line) and mean values (cross). Numbers in brackets represent the number of proteins in the corresponding category. The letters represent significant differences (*p* < 0.05, Kruskal–Wallis and Conover’s test, *p* < 0.05). For details, see Supplementary Table S1.

Nine plants per experimental condition were randomly selected, and all their seeds were homogenized and extracted. The proteome analysis of collected seeds provided the identification and quantitation of 3119 and 1796 proteins, respectively. The functional analysis of the corresponding *Arabidopsis* orthologs showed that the most numerous categories were protein metabolism, carbohydrate metabolism enzymes (CAZymes), stress-related proteins, and RNA metabolism, representing more than 900 proteins in total (Figure 3a). The seed composition based on the estimated protein abundances showed that the majority of the seed proteome was formed by storage proteins (76%), proteins of lipid metabolism (6%), stress response (5%), protein metabolism (4%), and CAZymes (3%) (Figure 3b). Next, the effect of salicylic acid treatment on seed proteome composition was compared (Figure 3c). Average changes in protein categories were relatively low, and there was no significant impact on the most abundant category (seed storage proteins). However, the comparison showed significant differences (*p* < 0.05) in most other categories, including protein metabolism, CAZymes, RNA metabolism, redox metabolism, transport, and lipid metabolism (Figure 3c).

### 2.4. Differentially Abundant Proteins Indicated a Decrease in Abscisic Acid Sensitivity

**Figure 4:**
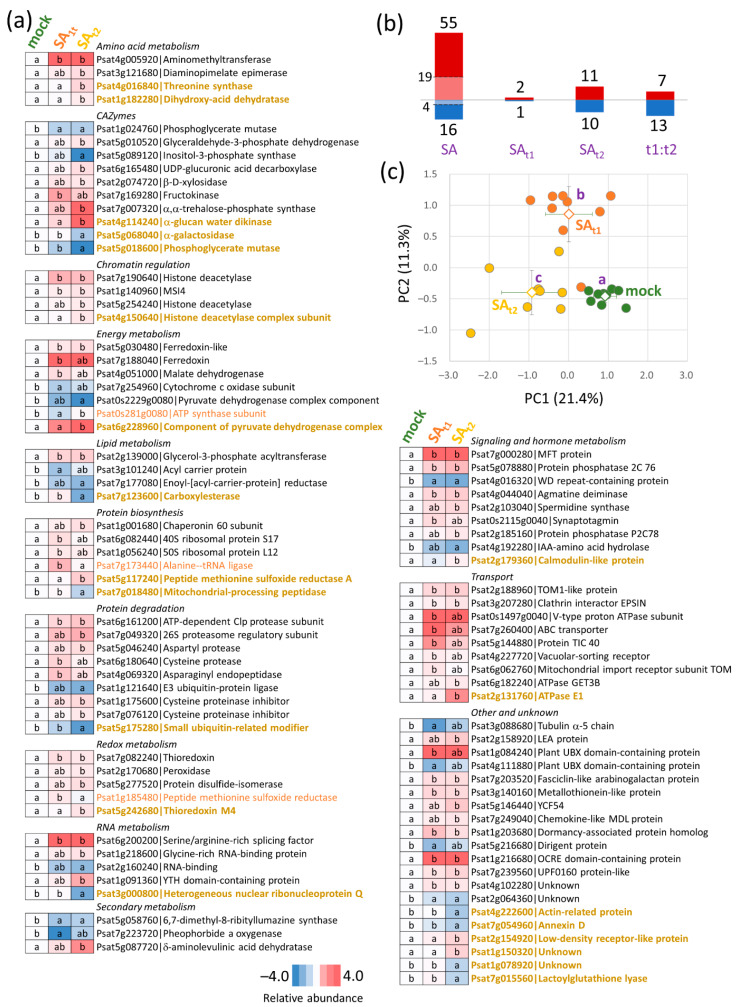
Differentially abundant proteins. (**a**) Heat maps represent the mean relative abundances of nine biological replicates; letters represent the results of Kruskal–Wallis and Conover’s test (*p* < 0.05); orange, SA_t1_-specific effect; beige, SA_t2_-specific effect. (**b**) Summary of the observed effects. SA_t1_, SA_t1_-specific effect; SA_t2_, SA_t2_-specific effect; SA, a similar effect in SA_t1_ and SA_t2_ seeds, the light shade indicates proteins with statistically significant response in both treatments; t1:t2, significant differences in SA_t1_ compared to SA_t2_. (**c**) PCA based on the profile of all differentially abundant proteins (*p* < 0.05, at least 1.4-fold change). Open shapes and dot lines represent means and standard deviations, respectively. The letters represent significant differences (Kruskal–Wallis and Conover’s test, *p* < 0.05). For details, see Supplementary Table S1.

The detailed analysis of protein abundances revealed 115 proteins that showed statistically significant differences in response to the salicylic acid treatment (Figure 4a–c; *p* < 0.05, Kruskal–Wallis and Conover’s test, *p* < 0.05). The PCA explained only 32% of the variability in the first two components but separated all three sample groups in the following order: mock—SA_t1_—SA_t2_ (Figure 4c). The pairwise comparison of the salicylic acid response showed 55 and 16 proteins with increased and decreased abundances, respectively. In accordance with PCA, the SA_t2_ treatment showed a higher impact on the proteome with 21 treatment-specific proteins and only three for SA_t1_. Finally, 20 proteins did not show a significant difference compared to the mock but were differentially abundant between SA_t1_ and SA_t2_ (Figure 4b, Table S1). The identified differentially abundant proteins represented a snapshot of different metabolic processes, including amino acid metabolism, chromatin regulation, carbohydrate metabolism, lipid metabolism, protein degradation, hormone metabolism, and signaling.

Differentially abundant proteins of interest that showed an increase in abundance in response to salicylic acid included trehalose 6-phosphate synthase that may promote seed filling [17], a lysine biosynthetic enzyme diaminopimelate epimerase with a putative role in salicylic acid-induced systemic acquired resistance [18], polyamine biosynthetic enzyme spermidine synthase and agmatine deiminase, an ortholog of protein phosphatase P2C78 which is a negative regulator of abscisic acid signaling and response to drought [19], MFT protein (regulates germination, mutant is hypersensitive to abscisic acid; [20]), two histone deacetylases and core histone-binding subunit MSI4 (AT2G19520) which ortholog promotes plant defense and flowering time in *Arabidopsis* [21], an ortholog of serine/arginine-rich splicing factor AT5G52040 mutation of which increases sensitivity to salt stress and abscisic acid [22], asparaginyl endopeptidase involved in processing of seed storage proteins, and an ortholog of glycine-rich RNA-binding protein RZ1A (overexpressors displayed earlier germination and better seedling growth under cold stress; [23]). Proteins that showed a decrease in abundance in response to salicylic acid included an ortholog of WD repeat-containing protein VIP3 (mutant shows early flowering; [24]), auxin activating enzyme that hydrolyzes IAA-amino acid conjugates, and an ortholog of a key enzyme in myo-inositol biosynthesis pathway AT2G22240 (inositol-3-phosphate synthase, mutant plants are compromised in resistance to pathogens; [25]). In summary, salicylic acid treatment induced the accumulation of proteins related to the abiotic stress response that may attenuate abscisic acid sensitivity. Interestingly, the comparison with the previously identified hormone-responsive proteins confirmed the putative role of abscisic acid signaling. In total, 32 matching orthologs were listed in the database, and 20 were proteins found in response to abscisic acid (Table S2; [26]).

### 2.5. Seeds of Plants Treated with Salicylic Acid Showed a Higher Accumulation of Storage Lipids

**Figure 5:**
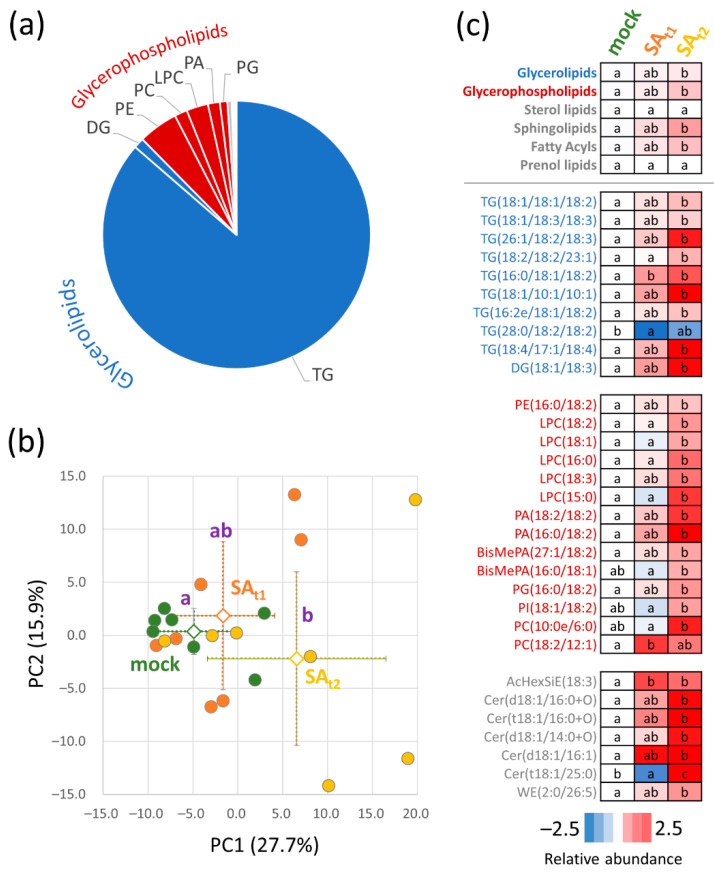
Lipidome profile of seeds. (**a**) The estimated seed lipid composition of mock-treated plants and corresponding PCA analysis (**b**). Based on the abundance of 250 detected lipids. Open shapes and dot lines represent means and standard deviations, respectively. (**c**) Statistically significant differences in lipid composition. Lipid classes and individual lipids are sorted according to the class and estimated abundance. Letters indicate statistically significant differences (*p* < 0.05, Kruskal–Wallis and Conover’s test, *p* < 0.05). Results are based on seven biological replicates. Cer, ceramides; DG, diglyceride; LPC, lysophosphatidylcholine; PA, phosphatidic acid; PC, phosphatidylcholine; PE, phosphatidylethanolamine; PG, glycerophosphoglycerols; PI, phosphatidylinositol; SiE, sitosterol esters; TG, triglyceride; WE, wax ester. For details, see Supplementary Table S2.

First, the quality of the lipid extracts was evaluated by TLC. Lipid extracts were spotted on a TLC plate, separated, and stained with Coomassie. Two sets of biological replicates showed a lower extraction efficiency, and these samples were excluded from the analysis. The remaining seven biological replicates were analyzed by direct infusion mass spectrometry, as described in Materials and Methods. The analysis of the seed lipidome provided reliable identification and quantitation of 250 lipid compounds. The seed lipidome was formed by storage glycerolipids and glycerophospholipids, representing, on average, 87.6 and 11.2% of the mock-treated seed lipidome (Figure 5a). The salicylic acid treatment showed an impact on the whole profile of the seed lipidome. However, significant differences were found only for SA_t2_ seeds (Figure 3b). The detailed analysis confirmed this trend (Figure 3c) and showed that salicylic acid treatment stimulated the accumulation of triglycerides and diglycerides (average increase of 19% compared to mock), glycerophospholipids (50% increase), sphingolipids (86% increase), and fatty acyls (56%). The results indicated an increase in the abundance of unsaturated triglycerides.

### 2.6. The Seed Metabolome Confirmed an Increase in Polyamine Production and Indicated the Accumulation of Phenolics

**Figure 6:**
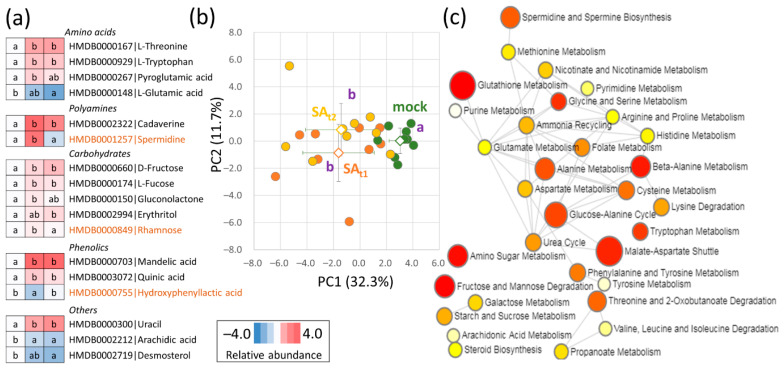
Differentially abundant metabolites in seeds of plants treated with salicylic acid. (**a**) Heat maps represent the mean relative abundances of nine biological replicates; letters represent the results of Kruskal-Wallis and Conover’s test (*p* < 0.05); orange, SA_t1_-specific effect; (**b**) PCA based on the profile of all differentially abundant metabolites (Student’s *t*-test, *p* < 0.05). Open shapes and dot lines represent means and standard deviations, respectively. The letters represent significant differences (*p* < 0.05, Kruskal–Wallis and Conover’s test, *p* < 0.05); (**c**) Enrichment analysis of metabolic pathways by MetaboAnalyst. Size and red color intensity indicate pathway significance. For details, see Supplementary Table S3.

The GC-MS analysis of the seed tissue metabolome provided quantitative data for 96 polar and semipolar metabolites. In contrast to proteomics and lipidomics data, the metabolome profile showed a high degree of biological variability and did not reveal significant differences between the SA_t1_ and SA_t2_ seeds (Figure 6a,b). Only 17 metabolites showed statistically significant and reproducible differences in response to salicylic acid treatment, including amino acids, carbohydrates, phenolics, and polyamines (Figure 6a, Table S3). The analysis of the overrepresentation of differentially abundant metabolites also pointed to a putative impact on glutathione metabolism, urea cycle, amino sugar metabolism, and beta-alanine metabolism (Figure 6c). Finally, the measured data were analyzed, and the fragment ions corresponding to salicylic acid were targeted and quantified. Comparison with the seeds of mock-treated plants did not show any impact on the salicylic acid content in the SA_t1_ seeds and only a mild and statistically insignificant increase in SA_t2_ (Table S3).

### 2.7. Integrative Analysis of Omics Data Indicated an Impact on the Citric Acid Cycle and Pyruvate Metabolism in Seeds of Plants Treated with Salicylic Acid

**Figure 7:**
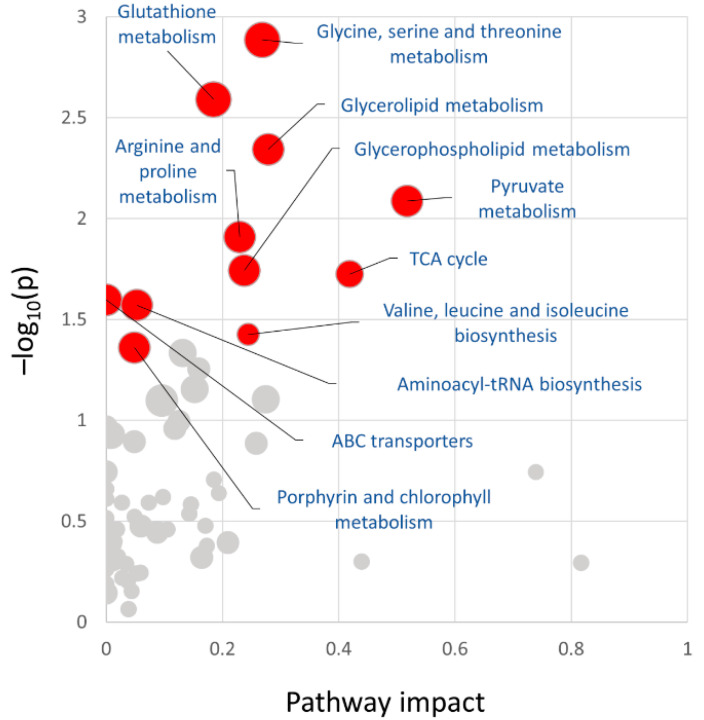
Metabolic pathway impacted by identified differentially abundant compounds. Proteins, lipids, and metabolites listed in Figure 4, Figure 5 and Figure 6 were analyzed using MetaboAnalyst. Highlighted pathways represent significantly enriched processes (*p* < 0.05).

Identified differentially abundant proteins, metabolites, and lipids were searched against annotated metabolic pathways in Arabidopsis thaliana using MetaboAnalyst integrative analysis. In total, the database matched 94 proteins (100%), 16 metabolites (94%), and 12 lipids (39%). The analysis provided additional evidence for the putative role of glutathione metabolism and amino acid metabolism and indicated a significant impact on the citric acid cycle and pyruvate metabolism (Figure 7).

## 3. Discussion

### 3.1. Positive Effects of Salicylic Acid Treatment on Seed Yield

The effect of salicylic acid on the yield in the field experiment was less than expected. A positive effect was found only for the large seed pods of the SA_t2_ plants and only with relaxed criteria (statistical significance threshold at *p* = 0.1). The application of salicylic acid before flowering (SA_t1_) promoted the growth of seed pods, but harmed seed production, resulting in a lower average weight of seeds per pod (Figure 2b–d). Interestingly, the total weight of the seeds collected from the large seed pods of SA_t1_ and SA_t2_ was comparable and was higher than that of the mock-treated plants by 12–15%. That was a lower effect than that observed in a similar experiment with rice (approximately 30% increase in yield; [27]). The employed 100 µM concentration was based on previous studies (e.g., [28]), and it is possible that a higher concentration of salicylic acid would have been needed for a more pronounced effect. It should be noted that the maximum daily temperatures reached 40 °C (Figure 1d). That is 15 °C above the optimal temperature limit [1], and it is likely that the protective effects of salicylic acid treatment were attenuated.

### 3.2. Polyamine Metabolism Could Correlate with the Increase in Number of Large Seed Pods

Proteome and metabolome data highlighted an increase in redox metabolism and a putative role for β-alanine and polyamines biosynthesis in response to salicylic treatment. The application of salicylic acid reportedly stimulates ROS metabolism (e.g., [29]), and the observed changes in thioredoxins (↑, Figure 4a), lactoylglutathione lyase (↓), peroxidase (↑), protein disulfide-isomerase could correspond to that. The biosynthesis of β-alanine would be in line with the expected role of this metabolite that accumulates in response to stress to protect plants from extreme temperatures [30]. However, the abundance of β-alanine was only slightly increased (14% in SA_t2_, Table S3), and the change in abundance was not statistically significant (*p* < 0.05). The increase in polyamine biosynthesis was supported by both proteome and metabolome data. Agmatine deiminase and spermidine synthase were more abundant (Figure 3a), and, in agreement, polyamines were also more abundant, including spermidine (SA_t1_), cadaverine (SA_t1_, SA_t2_), and putrescine (SA_t1_, Table S3, *p* < 0.1). There is evidence that polyamines protect reproductive tissues during stress exposure and that external application of polyamines could accelerate flowering [7]. The accumulation of polyamines was not specific to SA_t2_ seeds. In fact, it was more significant in the seeds of SA_t1_ plants, indicating that the polyamine levels could correlate with the observed increase in the number of large seed pods (Figure 2b).

### 3.3. Putative Protein Candidates Responsible for a Better Performance of SA_t2_ Plants

The average dry weight of seeds per pod was the highest for SA_t2_ plants (Figure 2c). In order to identify putative candidates responsible for the observed increase in yields, compounds that showed significant differences in abundance compared to mock and SA_t1_ were reviewed. The most promising protein candidate was an ortholog of heterogeneous nuclear ribonucleoprotein Q (↓, Figure 4a). In *Arabidopsis*, a mutation in the corresponding gene *LIF2* resulted in early flowering [31] and a disbalance in salicylic acid response genes [32]. A differentially abundant enzyme with a putative role in seed development was threonine synthase (↑, Figure 4a). It is reportedly accumulated at the late stage of seed development, and its activity competes with that of cystathionine γ-synthase and balances the threonine and methionine equilibrium [33]. Its higher abundance in SA_t2_ seeds could correlate with a higher number of well-developed seeds in SA_t2_ pods compared to those of mock and SA_t1_ plants. However, the pool of threonine was significantly higher in both SA_t1_ and SA_t2_ seeds (Figure 5a). Methionine was less abundant in SA_t1_, but the difference was not statistically significant (*p* < 0.05; Table S3). A promoted seed development could also negatively correlate with the abundance of a CAZyme α-galactosidase (↓, Figure 4a). It has a putative role in the loosening and expansion of the cell wall, a process that is attenuated during the cell rigidification at the late stage of seed development [34]. Proteins that could play a role in improved stress tolerance and promoted seed development in SA_t2_ plants included a regulator of starch mobilization α-glucan water dikinase (↑, Figure 4a; protein accumulation was positively correlated with heat stress in seeds of barley; [35]) and an ortholog of histone deacetylase complex subunit AT2G45640 (↑; mutant hypersensitive to salt; [36]). Finally, calmodulin accumulation (↑, Figure 4a) likely represented a response to salicylic acid [37], and it is well in line with the documented key role of calmodulin in the abiotic stress signaling cascade [38,39].

### 3.4. Putative Lipid Candidates Responsible for a Better Performance of SA_t2_ Plants

The differentially abundant lipids represented only 5.5% of the mock-treated seed lipidome (Table S2), but the lipidome profiling pinpointed several candidates of interest. The lipidome showed a significant increase in ceramide abundance, including a SA_t2_-specific accumulation of cer(t18:1/25:0). That could coincide with a lower abundance of inositol-3-phosphate synthase (the corresponding *Arabidopsis* mutant showed a higher abundance of ceramide and hydroxyceramide levels; [40]). The seeds of SA_t2_ plants showed a significant increase in the abundance of lysophosphatidylcholines (LPC, Figure 5c). These lipids could play a role in signaling through the production of lisophosphatidic acid [41], and that could coincide with an increase in the abundance of phosphatidic acids (Figure 5c). However, it is more likely that the observed increase in the abundance of LPCs is correlated with a higher level of triglycerides [42]. A recent study showed that PI(18:1/18:1) attenuated cytotoxic stress and stress signaling in mammalian cells through the activation of stress-activated kinases, the unfolded protein response, and autophagy [43]. It is tempting to speculate that a similar role could be found for PI(18:1/18:2), which was more abundant in the seeds of SA_t2_ plants (Figure 5c).

## 4. Materials and Methods

### 4.1. Plant Material and Cultivation

*Pisum sativum* (cv. Kudrnac) seeds were obtained from SEMO a.s., Smržice, Czechia. Seeds were without any chemical treatment, and only homogenous uniform seeds without any signs of damage were used for the experiment. All plants used for the study grew in the private experimental field plot in the South Moravian Region, Czechia (49°02′ N 17°33′ E; 195 m a.s.l.). Sowing was done after the rainfall on June 1, 2022, and plants were grown in a natural environment without any additional treatment. After 35 days, the field was divided into three blocks (mock, SA_t1_, SA_t2_), each containing approximately 100 plants. For five weeks, plants were weekly sprayed with 1.5 L water solution supplemented with 0.025% (*v*/*v*) Silwet L-77 (mock), or 100 µM salicylic acid and Silwet (final concentration as for the mock; SA_t1_). The third set was treated as the mock for two weeks, followed by four weeks with salicylic acid treatment (SA_t2_). The whole design is illustrated in Figure 1c. In total, 75 plants per treatment were collected. For omics analyses, nine plants per experimental condition (*n* = 9) were randomly selected, and all their seeds were homogenized and extracted as described in the following chapters.

### 4.2. Proteome Analysis

Aliquots of homogenized seeds (approximately 100 mg of homogenized tissue per biological replicate) were extracted for omics analyses as described previously [35,44,45,46], and portions of samples corresponding to 5 µg of peptide were analyzed by nanoflow reverse-phase liquid chromatography-mass spectrometry using a 15 cm C18 Zorbax column (Agilent, Santa Clara, CA, USA), a Dionex Ultimate 3000 RSLC nano-UPLC system, and the Orbitrap Fusion Lumos Tribrid Mass Spectrometer equipped with a FAIMS Pro Interface (Thermo Fisher, Waltham, MA, USA). All samples were analyzed using FAIMS compensation voltages of −40, −50, and −75 V, and a pooled sample was screened across compensation voltages using a 5 V step gradient. The measured spectra were recalibrated, filtered (precursor mass—350–5000 Da; S/N threshold—1.5), and searched against the *P. sativum* protein database (GCA_900700895, [47]) and common contaminants databases using Proteome Discoverer 2.5 (Thermo, algorithms SEQUEST and MS Amanda [48]). The quantitative differences were determined by Minora, employing precursor ion quantification followed by normalization (total area) and calculation of relative peptide/protein abundances.

### 4.3. Metabolome Analysis

Metabolite fraction was derivatized and measured using a Q Exactive GC Orbitrap GC-tandem mass spectrometer and Trace 1300 Gas chromatograph (Thermo) as described in [49,50,51]. In brief, samples were derivatized by 10 μL of methoximation solution (40 mg of methoxyamine hydrochloride in 1 mL of pyridine) and incubated for 90 min at 30 °C with continuous shaking. After the incubation, 40 μL of silylation solution (*N*-methyl-*N*-trimethylsilyltrifluoroacetamide) was added, and the mixture was incubated for 30 min at 37 °C with continuous shaking. Metabolites were injected onto the TG-5SILMS GC column (Thermo Fisher, 30 m × 0.25 mm × 0.25 μm), separated with a 28 min gradient (70 to 320 °C), and ionized using the electron ionization mode (electron energy 70 eV, emission current 50 μA, transfer line and ion source temperature 250 °C). Data were analyzed by Compound Discoverer 3.3 (Thermo; peak detection settings—5 ppm; TIC threshold—50,000; S/N threshold—3) and searched against NIST2014, GC-Orbitrap Metabolomics library, and in-house library. Only metabolites that met stringent identification criteria (score > 90 and ΔRI < 5%) were included in the final list of identified compounds. The salicylic acid pool was analyzed by comparing extracted chromatograms corresponding to fragments of its 2TMS derivative (267.0864, 193.0675, and 249.0757) at the expected retention determined by its spiked deuterated analog [^2^H_4_]salicylic acid (Olchemim, Czech Republic) employing Skyline 19.1 [52].

### 4.4. Lipidome Analysis

The lipid fraction was dried by vacuum centrifugation, redissolved in 200 µL isopropanol/methanol/tert-butyl methyl ether 4/2/1 (*v*/*v*/*v*) with 20 mM ammonium formate, and analyzed by direct infusion using a Triversa Nanomate (Advion Biosciences, Ithaca, NY, USA) nanoelectrospray source as described previously [53,54]. The acquired profile spectra were analyzed using FreeStyle 1.7 and LipidSearch 4.2 (Thermo Fisher; precursor tolerance—5 ppm; product ion tolerance—10 ppm; m-Score threshold—2.0).

### 4.5. Data Analysis and Statistics

The reported statistical tests were generated and implemented as follows using default and recommended settings unless otherwise indicated. The reliability of protein identifications was assessed in Proteome Discoverer 2.5 (Thermo Fisher Scientific). The Student’s *t*-test was calculated using MS Excel. For the Kruskal-Wallis test, the Real Statistics Resource Pack software for MS Excel (Release 6.8; Copyright 2013–2020; Charles Zaiontz; www.real-statistics.com; accessed on 15 February 2023). PCAs were performed in MetaboAnalyst 5.0 using mean centering, and the employed data filtering is indicated in the corresponding figure legends [55]. Significant differences refer to *p* < 0.05 unless otherwise stated. Protein functional annotations were obtained by using the UniProt database (https://www.uniprot.org; accessed on 15 February 2023) and updating ProteoMap annotations (http://bionic-vis.biologie.uni-greifswald.de/; accessed on 15 February 2023; [56]).

## 5. Conclusions

This work provided the first insight into the molecular composition of *P. sativum* seeds grown in plants treated with salicylic acid. The outlined experiments demonstrated that the application of salicylic acid promoted the yield of *P. sativum* grown under heat stress and indicated that the timing of the treatment was a critical factor. The composition of the proteome, metabolome, and lipidome of the seeds collected from treated plants was not drastically different from that of mock-treated ones. Furthermore, salicylic acid did not accumulate in the seeds of treated plants, indicating that the treatment did not result in traceable changes that could potentially limit the market value of the harvest. Despite the observed high similarity to the seeds of mock-treated plants, the analysis of SA_t1_ and SA_t2_ seeds provided a snapshot of molecular processes that pointed to putative circuits of improved resilience. Integrating the omics data showed evidence of polyamine production and pointed to the attenuation of abscisic acid signaling, modulations of ROS metabolism, and accumulation of lysophosphatidylcholines. This work provided novel targets for further analyses that should be explored in the future using mutant lines and time-series experiments following seed development.

## Data Availability

Data supporting the results are provided in the tables in the Supplementary Materials. The mass spectrometry proteomics data have been deposited to the ProteomeXchange Consortium via the PRIDE [57] partner repository with the dataset identifier PXD040506.

## Supplementary Materials

The following supporting information can be downloaded at: https://www.mdpi.com/article/10.3390/ijms24065454/s1.
